# SERS Response of Graphene Oxide on Magnetron-Sputtered Gold Films

**DOI:** 10.3390/nano15181438

**Published:** 2025-09-18

**Authors:** Grazia Giuseppina Politano

**Affiliations:** Department of Environmental Engineering, University of Calabria, 87036 Rende, Italy; grazia.politano@unical.it

**Keywords:** graphene oxide, thin films, gold, SERS, Raman, enhancement

## Abstract

Graphene oxide (GO) is a two-dimensional material with interesting optical properties, widely studied for its potential in ultrasensitive detection of substances and prospective optoelectronic properties. In this study, GO thin films were deposited onto gold layers obtained by direct current (DC) magnetron sputtering, and their Raman scattering response was evaluated. While most Surface Enhanced Raman Scattering (SERS) applications rely on gold nanoparticles, the use of magnetron-sputtered gold films remains relatively underexplored. GO layers were deposited by dip-coating and characterized by micro-Raman spectroscopy and scanning electron microscopy (SEM). Raman spectra of GO on Au samples show a clear enhancement of signal intensity compared to GO on glass, with well-preserved D and G bands and no evident structural degradation.

## 1. Introduction

Graphene oxide (GO) [1] is a widely studied carbon-based material [2], typically produced by chemical oxidation of graphite using methods such as the Hummers procedure [3]. The introduction of oxygen-containing groups (epoxide, hydroxyl, carboxyl, carbonyl) increases the interlayer spacing and enhances the hydrophilicity of the material, allowing for its dispersion in water and exfoliation into single- or few-layer sheets [4].

The chemical structure of GO has been extensively studied in previous works using X-ray photoelectron spectroscopy (XPS) [5]. Typically, the C 1 s spectrum of GO exhibits peaks associated with C=C/C–C (~284.8 eV), C–O (~286.6 eV), and C=O (~288.4 eV) [5].

GO is often combined with noble metals [6], particularly gold (Au) [7], to form nanocomposites with interesting properties for applications in catalysis [8], sensing [9], and biomedicine [10]. Among these, GO on Au systems have been extensively studied in the context of surface-enhanced Raman spectroscopy (SERS) [11], a technique that amplifies Raman signals by exploiting the plasmonic effects of metallic surfaces. SERS [12] allows the detection of analytes at very low concentrations and is used in several research fields, including chemistry, materials science, and biosensing [13]. SERS has also been recently applied to chiral discrimination, for instance, through the use of engineered two-dimensional materials with intrinsic chirality, such as TaS_2_-based chiral superlattices, which enable direct, label-free enantiospecific detection without the need for molecular selectors [14].

In most cases, Au is used in the form of nanoparticles [15] or nanostructured surfaces [16]. Several studies report high SERS enhancement factors (EFs), typically reaching values between 10^3^ [17] and 10^8^ [18], thereby demonstrating the strong potential of the GO/gold nanoparticle (GO/AuNP). However, the use of Au thin films deposited by magnetron sputtering as SERS substrates remains largely unexplored, despite the potential advantages of this method. Magnetron sputtering [19] is a reliable technique for the deposition of metallic and compound thin films. It offers good control over thickness, surface morphology, and film uniformity, and has been continuously optimized to improve deposition rates, ionization of sputtered species, and overall stability [20].

The present work investigates the SERS effect of GO deposited on Au thin films prepared by DC (direct current) magnetron sputtering. A previous study by the author [21] investigated the optical properties of GO films deposited on magnetron-sputtered gold using ellipsometry. In contrast, the present work focuses exclusively on the Raman response of GO, highlighting its enhancement when deposited on magnetron sputtered Au thin films.

## 2. Materials and Methods

Au thin films with a nominal thickness of approximately 15 nm were deposited onto glass substrates by DC magnetron sputtering (Edwards Auto306 system, West Sussex, UK). The thickness of the gold film was determined by Variable Angle Spectroscopic Ellipsometry (VASE) using an ellipsometer (model J.A. M2000 F, Woollam Co., Lincoln, NE, USA) [22]. The deposition was carried out under an argon atmosphere at a pressure of 4.2 × 10^−2^ mbar, with a base vacuum of 10^−5^ mbar. A cathode power of 30 W was applied for 55 s. Glass substrates were previously cleaned using a piranha solution (a mixture of H_2_SO_4_ and H_2_O_2_) to ensure surface cleanliness and promote film adhesion. The continuity and morphology of gold thin films at similar thickness (~15 nm) are well documented in the literature. Gold films become continuous when thickness reaches 8 nm [23]. Moreover, the morphology and the continuity of the gold thin films were proved in the author’s previous work [21].

GO films were deposited onto the sputtered Au layers via dip-coating [24] using a custom-made setup, with a withdrawal speed of 0.33 mm/s. The GO dispersion (2 g/L in water, Punto Quantico, Rome, Italy) was used as received. The GO film thickness was determined using VASE and found to be approximately 8 nm [21].

The dimensions of the GO flakes were estimated through translational diffusion analysis using dynamic light scattering. Autocorrelation functions of the scattered light were obtained using a Brookhaven Instruments 2030AT digital correlator (Brookhaven Instruments, Nashua, NH, USA), which processed the photocurrent pulses generated by a 9863 A Thorn-EMI photomultiplier equipped with an integrated amplifier-discriminator.

Highly diluted aqueous GO dispersions were illuminated with a finely focused He-Ne laser beam (35 mW, model 127, Spectra Physics), and the scattered light was collected at a fixed scattering angle of 90°. The resulting autocorrelation data were analyzed using the CONTIN algorithm to perform Laplace inversion, allowing for the extraction of the flake size distribution.

**Figure 1:**
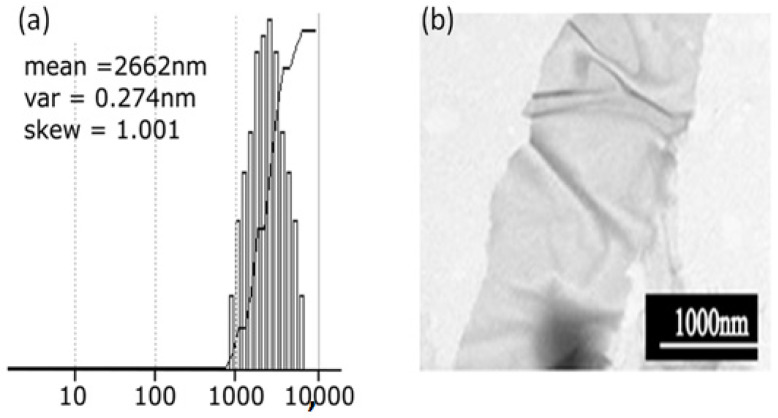
Particle size distribution of highly diluted graphene oxide (GO) flakes dispersed in water, obtained via dynamic light scattering. (**a**) and TEM image of GO flakes at high magnification, consistent with the morphology described in the supplier’s datasheet (**b**).

A Gaussian-like distribution was obtained, with flake sizes ranging approximately from 800 nm to 7 μm, as shown in Figure 1a. Moreover, based on the supplier specifications, GO flakes are planar structures with lateral dimensions on the micrometer scale and thicknesses in the nanometer range, as confirmed by Figure 1b.

Micro-Raman measurements of GO on glass and of GO on Au samples were performed using a Horiba-Jobin Yvon apparatus (model LabRam HR, Horiba, Darmstadt, Germany), consisting of a single grating spectrograph, a 1800 lines/mm holographic grating, an edge filter, and a 100× Mplan Olympus objective with a NA of 0.90. Spectra were excited by a source laser (532 nm) with a typical power of 50 mW, focused on a spot of about 1 μm diameter. In addition, to avoid structural changes due to laser heating an OD2 filter (1% allowed transmission) was applied, with an acquisition time of 1 s and 5 accumulations per spectrum.

Raman spectra were collected at ten different points on each sample to account for possible inhomogeneities.

Scanning Electron Microscopy (SEM) was performed using a FEI Quanta FEG 400 ESEM instrument (Fei, Eindhoven, The Netherlands), using an Everhart–Thornley detector at accelerating voltage of 10 kV.

## 3. Results and Discussion

### 3.1. Micro-Raman Measurements

**Figure 2:**
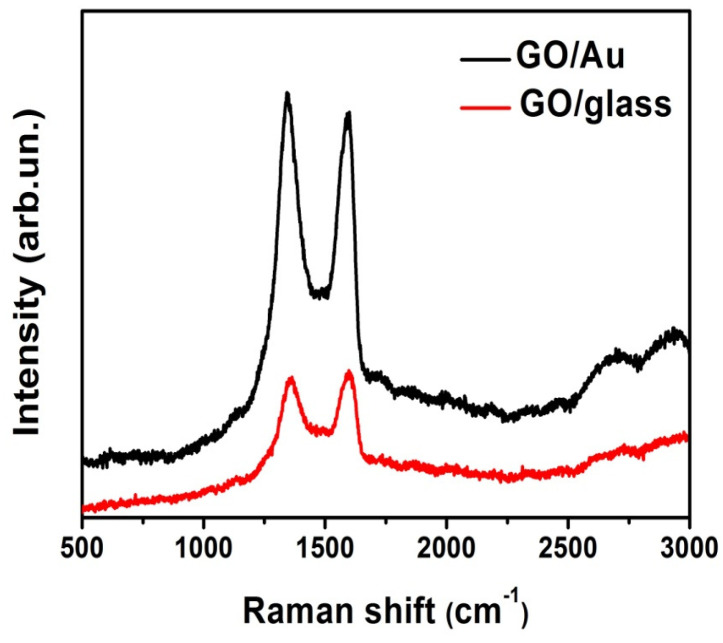
Representative Raman spectra of GO on glass (red curve) and of GO on Au (black curve) under low laser power conditions (OD2 filter (1% allowed transmission).

Micro-Raman spectroscopy was employed to characterize the deposited GO layers, as shown in Figure 2.

The Raman spectrum of GO deposited on a glass substrate (red line), measured at low laser intensity (OD2 filter, 1% transmission), reveals the characteristic D (~1365 cm^−1^) and G (~1595 cm^−1^) bands, commonly attributed to structural disorder and sp^2^-hybridized carbon domains in GO, respectively. Additionally, the 2D (~2640 cm^−1^) and D + G (~2912 cm^−1^) overtone bands are clearly visible [25].

The Raman spectrum of the GO on Au sample (black curve) closely resembles that of GO on glass. This observation suggests that the dip-coating process effectively preserved the structural features of GO without inducing noticeable chemical damage or degradation. Notably, the overall Raman signal intensity is higher for the GO on Au sample compared to GO on glass, indicating a strong interaction between the GO layers and the Au substrate. This enhancement can be attributed to SERS effects arising from the metallic substrate, which amplifies the Raman intensity of the GO film. The enhancement originates from the electromagnetic (EM) mechanism [26], in which the incident light excites localized surface plasmon resonances (LSPRs) [27] on the gold surface. These plasmonic excitations generate highly localized electric fields at the Au–GO interface, which significantly amplify the Raman scattering signals of the GO layer. This enhancement allows for a more sensitive detection of the vibrational modes of GO, even when deposited as ultrathin films. Moreover, previous research works have suggested that the interaction between GO and Au may include partial charge transfer at the interface, potentially contributing a chemical enhancement component to the overall SERS effect [28].

The Raman spectra collected across multiple regions of the GO on Au sample consistently show an I_D_/I_G_ intensity ratio greater than 1, indicating the presence of structural disorder and defective sp^2^ domains [29].

The Enhancement Factor (EF) is commonly expressed as follows [30].

(1) $$EF=\frac{I_{SERS}/\left(N_{SERS}\times P_{SERS}\times T_{SERS}\right)}{I_{Raman}/\left(N_{Raman}\times P_{Raman}\times T_{Raman}\right)}$$

where $I_{SERS}$ is the signal intensity measured on the SERS substrate and $I_{Raman}\;$ is the Raman signal intensity measured on the non-SERS substrate. The ratio *I_SERS_*/*I_Raman_* is normalized to the number of molecules (*N_SERS_* and *N_Raman_*), the excitation powers (*P_SERS_* and *P_Raman_*), and integration times (*T_SERS_* and *T_Raman_*) used for each acquisition.

The number of molecules *N* interacting with the laser can be estimated as the product of the illuminated area and the molecular surface density. Since the Raman and SERS measurements were conducted under identical optical conditions (i.e., same objective lens and laser spot size), it is rational to assume similar surface coverage for both substrates. As a result, *N_SERS_*~*N_Raman_*.

Moreover, identical laser powers and acquisition times were used in both measurements. Under these specific conditions, the normalization terms cancel out, and the EF simplifies to:

(2) $$EF=\frac{I_{SERS}}{I_{Raman}}$$

where *I_SERS_* is the SERS signal intensity of GO measured on Au substrates and *I_Raman_* is the Raman signal measured on glass substrate.

The estimated enhancement factor (EF ≈ 4) refers to the intensity ratio of the D-band in the Raman spectrum of GO on magnetron-sputtered gold compared to GO on glass, measured under the same laser power, acquisition time, and optical setup.

Although the estimated EF of ~4 is relatively modest compared to the values typically reported for gold nanoparticle-based SERS substrates, this result is still of significant interest. First, the use of magnetron-sputtered Au films avoids the need for complex synthesis routes, chemical stabilizers, or advanced nanostructuring techniques, which are often required for the fabrication of nanoparticle-based systems. Another important advantage of using magnetron-sputtered Au films as SERS platforms lies in the high reproducibility of the deposition process itself. Physical vapor deposition techniques such as DC magnetron sputtering allow for precise control over key parameters including deposition time, cathode power, working pressure, and film thickness, ensuring a high degree of uniformity and consistency across different samples and experimental runs. This contrasts with the often variable nature of colloidal nanoparticle synthesis, where batch-to-batch differences, aggregation effects, and surface chemistry inconsistencies can strongly affect the reproducibility of the SERS response.

### 3.2. SEM Measurements

**Figure 3:**
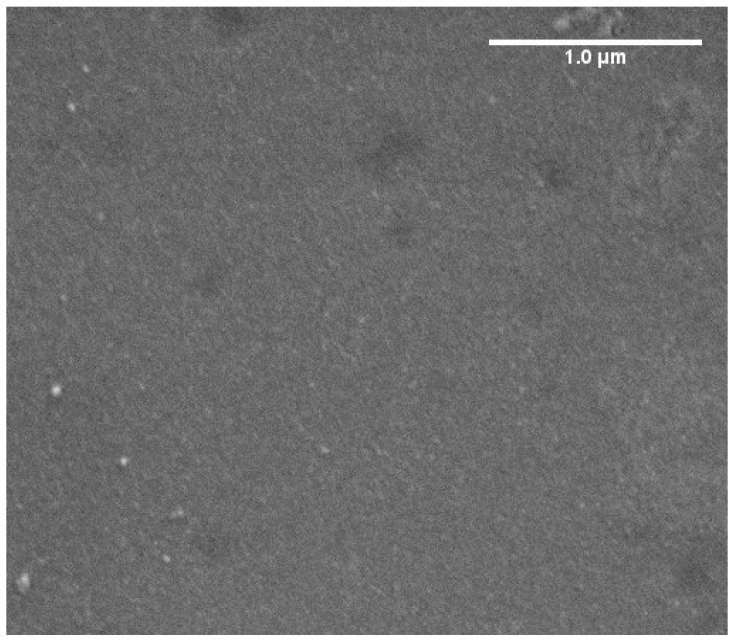
SEM image of a typical region of GO films dip-coated on magnetron sputtered gold thin film.

SEM image in Figure 3 shows that at the spatial scale relevant to Raman analysis (~1 μm) the morphology of the samples appears rather uniform. Given that the Raman spot size is approximately 1 μm, the enhancement observed across different points is consistent, with no significant local variation detected. This suggests that, despite some macroscopic inhomogeneities, the GO film ensures reasonably uniform interaction with the underlying Au substrate at the scale probed by micro-Raman spectroscopy.

## 4. Conclusions

In this work, the Raman enhancement of GO films deposited by dip-coating onto Au thin films obtained by DC magnetron sputtering was studied. Although the estimated EF ≈ 4 is relatively low compared to values reported in the literature for gold nanoparticles (~10^3^), the result is still relevant.

The use of DC magnetron sputtering allows precise control of the deposition parameters, such as power, pressure, and time, leading to uniform and reproducible gold films. The Raman measurements collected in different areas of the sample confirmed the stability and homogeneity of the GO–Au interface at the microscale. This method is simple and reproducible, and does not require complex synthesis. For this reason, it can represent a valid approach for researchers who are approaching SERS applications for the first time or who do not have access to advanced nanoparticle fabrication techniques.

## Data Availability

The data are contained inside the manuscript.

## Abbreviations

The following abbreviations are used in this manuscript:

GO	Graphene oxide
Au	Gold
SERS	Surface Enhanced Raman Scattering
LSPRs	Localized surface plasmon resonances
